# Association between lactate dehydrogenase and 28-day all-cause mortality in patients with non-traumatic intracerebral hemorrhage: A retrospective analysis of the MIMIC-IV database

**DOI:** 10.17305/bb.2024.11189

**Published:** 2024-11-06

**Authors:** Jia Hui Feng, Ren Jie Liu, Xuan Chen

**Affiliations:** 1 Department of Neurovascular Disease, The First Hospital of Jilin University, Changchun, China

**Keywords:** Lactate dehydrogenase, non-traumatic intracerebral hemorrhage, retrospective, MIMIC

## Abstract

Lactate dehydrogenase (LDH), a nonspecific inflammatory biomarker, has been used in the assessment of acute myocardial infarction, acute hepatitis, acute lung injury, and other severe diseases. However, no studies have evaluated the prognostic value of LDH in patients with non-traumatic intracerebral hemorrhage (ICH). This cohort study aims to assess the association between LDH levels and 28-day all-cause mortality in patients with non-traumatic ICH. Data for this retrospective cohort analysis were obtained from the MIMIC-IV (v2.2) database, and the study included patients with non-traumatic ICH as defined by the International Classification of Diseases, 9th and 10th editions. Patients were categorized into four distinct groups based on their LDH levels. The primary outcome of interest was the 28-day mortality rate. To analyze these associations and assess the consistency of interactions, subgroup analyses, Cox regression analysis, Kaplan–Meier (K–M) curves, and nonlinear analysis were conducted. A total of 406 patients with non-traumatic ICH were enrolled in the study and were divided into quartiles based on LDH levels. The K–M curve indicated that the 28-day all-cause mortality rate of patients in the Q4 group (LDH > 287.25) was significantly higher than in the Q1 (LDH < 194.7) (*P* < 0.001) and Q2 (194.7 < LDH < 233.0) (*P* < 0.001) groups, though not significantly different from Q3 (*P* ═ 0.140). Multivariate Cox proportional hazards analysis revealed that patients in the highest LDH quartile had a significantly increased risk of mortality compared to those in the lowest quartile across three models: unadjusted [HR, 3.401; 95% CI, 1.719–6.731; *P* < 0.001], partially adjusted [HR, 2.422; 95% CI, 1.211–4.846; *P* ═ 0.012], and fully adjusted [HR, 3.054; 95% CI, 1.522–6.126; *P* ═ 0.002]. Restricted cubic spline (RCS) models revealed an L-shaped association between LDH levels and the 28-day all-cause mortality rate, indicating a nonlinear relationship (*P* < 0.001). No significant interactions were observed between LDH levels and other factors in the subgroup analyses (all *P* for interaction > 0.05). Our findings indicate a significant association between 28-day all-cause mortality and LDH levels in patients with non-traumatic ICH. Specifically, patients with elevated LDH levels within the first 24 h of ICU admission are at a higher risk of mortality.

## Introduction

Intracerebral hemorrhage (ICH) accounts for approximately 10%–15% of all strokes and is associated with high mortality and morbidity, resulting in poor outcomes. This includes hypertensive cerebral hemorrhage, spontaneous subarachnoid hemorrhage, and hemorrhage of autovascular causes [1, 2]. Patients with ICH typically present with an abrupt onset of focal neurologic signs [3]. Due to the high incidence of disabilities, recurrent stroke, cognitive decline, and systemic vascular disorders among survivors, ICH holds significant importance among neurologic conditions. Given the critical condition of these patients, there is an urgent need for a non-invasive and cost-effective testing method in clinical practice to identify individuals at higher risk of mortality and to prevent adverse outcomes [4].

**Figure 1.**
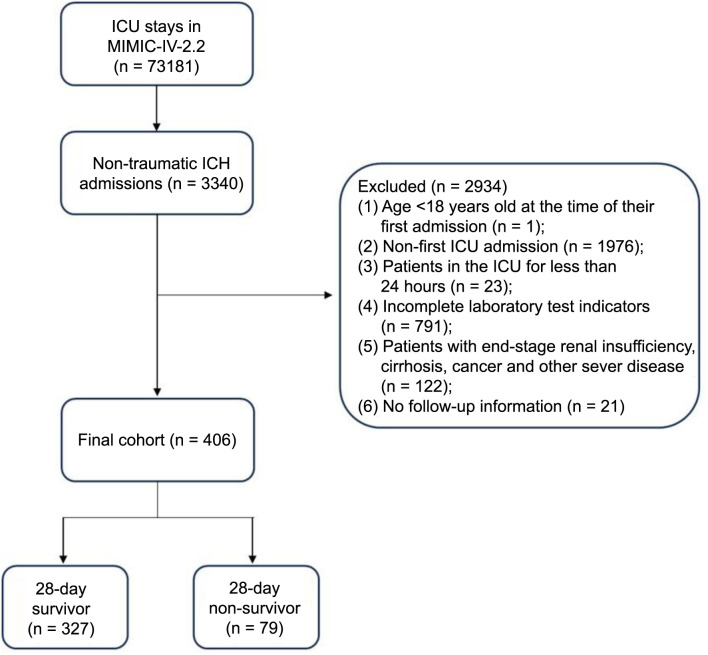
**Inclusion/exclusion criteria.** MIMIC: Medical Information Mart for Intensive Care; ICU: Intensive care unit; ICH: Intracerebral hemorrhage.

Lactate dehydrogenase (LDH) is a crucial enzyme involved in the anaerobic metabolic pathway and is predominantly located in the cytoplasm and mitochondria of various tissues, including the brain, heart, liver, and lungs. In response to tissue damage caused by diseases or pathological mechanisms, LDH is released into the extracellular space, resulting in elevated serum levels. This elevation is observed in conditions such as acute myocardial infarction, acute hepatitis, and acute lung injury [5]. LDH serves as a nonspecific inflammatory biomarker [6]. Consequently, this cohort study aims to investigate the relationship between LDH levels and the 28-day survival rate in patients with non-traumatic ICH.

## Materials and methods

### Study population

This study was a retrospective observational cohort analysis involving longitudinal follow-up of patients. The Medical Information Mart for Intensive Care-IV (MIMIC-IV-2.2) is a comprehensive and widely used database developed and maintained by the MIT Computational Physiology Laboratory. The database includes records of over 50,000 adult patients between 2008 and 2019. The MIMIC-IV database encompasses a wealth of information, including demographics, vital signs, and test results. Patients diagnosed with non-traumatic ICH were included in this study based on the 9th and 10th editions of the International Classification of Diseases.

### Selection criteria

The MIMIC-IV database recorded a total of 523,740 admissions. Hospital admission records for patients with non-traumatic ICH were extracted using the International Classification of Diseases, 9th Revision (ICD-9) code 431, and the International Classification of Diseases, 10th Revision (ICD-10) codes I610, I611, I612, I613, I614, I615, I616, I618, and I619, resulting in a total of 3340 patients included. The exclusion criteria were as follows: (1) patients who were under 18 years old at the time of their first admission; (2) patients with multiple ICU admissions, with only data from the first admission being considered; (3) patients with severe conditions such as end-stage renal insufficiency, cirrhosis, or cancer; (4) patients who were hospitalized in the ICU for less than 24 h; and (5) patients with incomplete laboratory test results. Ultimately, a total of 406 patients met the inclusion criteria and were categorized into four groups based on the quartiles of LDH levels (Figure 1).

### Variable extraction

In this study, data extraction was performed using Navicat Premium software (version 16) with Structured Query Language (SQL). Data extracted from the MIMIC-IV database within the first 24 h of ICU admission included age, gender, and race. Additional relevant data, including laboratory test results, clinical outcomes, and comorbidities, were also obtained. All laboratory parameters extracted from the MIMIC-IV (2.2) database were measured at the first instance after ICU admission. Follow-up began on the date of ICU admission and concluded on the date of death.

### Primary outcome

The primary outcome of this study was the 28-day mortality rate following ICU admission. In-hospital information was recorded by the hospital department, while out-of-hospital information was recorded by the Social Security Bureau.

### Ethical statement

To protect patient privacy, all personal information was deidentified, with random codes used to replace patient identifiers. Consequently, informed consent and ethical approval were not required. To access the database, the authors (Jiahui Feng) obtained the necessary authentication and subsequently extracted variables, including demographics, comorbidities, laboratory results, laboratory indices, and discharge status, from the MIMIC-IV database for this study (certification number: 62270682).

### Statistical analysis

The Kolmogorov–Smirnov test was employed to assess the normality of continuous variables. Data were presented as mean and standard deviation (SD) for normally distributed continuous variables, median and interquartile range (IQR) for non-normally distributed continuous variables, and as counts and percentages for categorical variables. Continuous variables were compared using Student’s *t*-test or one-way ANOVA, while categorical variables were examined using Fisher’s exact test or the chi-squared test. Kaplan–Meier (K-M) curves were plotted to evaluate mortality distributions among patients with varying LDH levels. Selected variables were incorporated into both univariate and multivariate Cox proportional hazards models to explore the association between LDH levels and 28-day all-cause mortality. Restricted cubic spline (RCS) analysis was used to visually depict the relationship between LDH levels and the risk of 28-day mortality. Additionally, subgroup analyses were conducted based on age, gender, Charlson Comorbidity Index, SAPS II score, and APS III score.

## Results

### Baseline demographic and clinical characteristics

A total of 406 patients with non-traumatic ICH were enrolled in this study. Baseline characteristics of patients with non-traumatic ICH, divided according to LDH quartiles, are presented in Table 1. Patients in the higher LDH quartile had elevated levels of white blood cells (WBCs) and anion gap, along with lower levels of bicarbonate, calcium, and albumin. Furthermore, they exhibited a higher prevalence of congestive heart failure and chronic pulmonary disease compared to the lower quartile. Table 2 presents the characteristics of patients with varying outcomes at 28 days. Factors such as age, Charlson Comorbidity Index, SAPS II score, APS III score, red blood cells (RBCs), WBC, mean corpuscular volume (MCV), red cell distribution width (RDW), hemoglobin, hematocrit, blood urea nitrogen (BUN), potassium, calcium, and albumin were found to be closely associated with patient outcomes (*P* < 0.05).

**Table 1:** Characteristics and outcomes of participants categorized by LDH

**Variables**	**Q1(<194.7, *n* ═ 101)**	**Q2(194.7∼233.0, *n* ═ 102)**	**Q3 (233.0∼287.3, *n* ═ 102)**	**Q4(>287.25, *n* ═ 101)**	***P* value**
Female, *n* (%)	43 (10.6%)	42 (10.3%)	30 (7.4%)	49 (12.1%)	**0.044**
Age (years)	69 (57, 79)	69 (57, 79)	67.5 (56, 78)	68 (55, 80)	0.923
Race, *n* (%)					0.090
White	67 (16.5%)	55 (13.5%)	60 (14.8%)	49 (12.1%)	
Yellow	3 (0.7%)	1 (0.2%)	2 (0.5%)	2 (0.5%)	
Black	10 (2.5%)	15 (3.7%)	10 (2.5%)	7 (1.7%)	
Other	21 (5.2%)	31 (7.6%)	30 (7.4%)	43 (10.6%)	
Charlson index	6 (4, 7)	6 (4.25, 7.75)	6 (5, 8)	6 (4, 8)	0.482
*Severity score*					
SAPS II	29 (23, 39)	31 (23.25, 39.75)	31 (24.25, 37.75)	34 (27, 42)	0.125
APS III	35 (26, 49)	41 (28.25, 51)	43.5 (31.5, 62.75)	46 (37, 63)	**<0.001**
GCS	15 (14, 15)	15 (14, 15)	15 (14, 15)	15 (14, 15)	0.620
*Commorbidities, n (%)*					
Myocardial infarct	7 (1.7%)	5 (1.2%)	2 (0.5%)	11 (2.7%)	0.060
Congestive heart failure	5 (1.2%)	11 (2.7%)	15 (3.7%)	18 (4.4%)	**0.032**
Chronic pulmonary disease	7 (1.7%)	7 (1.7%)	18 (4.4%)	19 (4.7%)	**0.008**
Mild liver disease	4 (1%)	7 (1.7%)	7 (1.7%)	8 (2%)	0.690
Diabetes	8 (2%)	5 (1.2%)	5 (1.2%)	5 (1.2%)	0.734
Laboratory tests					
RBC (10^12^/L)	4.38 (4, 4.85)	4.37 (3.895, 4.87)	4.335 (3.885, 4.6975)	4.3 (3.79, 4.82)	0.753
WBC (10^9^/L)	9.4 (7.1, 11.9)	9 (7.5, 12.325)	10.35 (8.25, 12.775)	10.7 (8.2, 13.8)	**0.012**
Platelet (K/µL)	216 (173, 260)	221 (174.5, 270.5)	201 (149.25, 264)	209 (164, 269)	0.353
MCH (pg)	30.2 (28.4, 31.7)	30.2 (28.6, 31.675)	30.6 (28.95, 32.175)	30.2 (28.6, 31.9)	0.658
MCHC (g/L)	33.5 (32.6, 34.4)	33.4 (32.3, 34.2)	33.5 (32.4, 34.4)	33 (32.4, 34)	0.209
MCV (fL)	90 (87, 92)	91 (87, 94.75)	91 (87, 95)	91 (87, 97)	0.230
RDW (%)	13.4 (13, 14.3)	13.5 (13.1, 14.475)	13.6 (13.1, 14.3)	13.8 (13.3, 14.4)	0.212
PT (S)	28.2 (25.8, 31.3)	27.85 (24.95, 30.975)	27.1 (25.125, 31.375)	27.8 (25.1, 31.6)	0.838
Hemoglobin (g/dL)	13.1 (12, 14.5)	13.2 (11.7, 14.1)	13.1 (11.925, 14.175)	13 (11.8, 14.5)	0.900
Hematocrit (%)	39.1 (36, 42.8)	39.25 (34.825, 42.675)	39.05 (35.75, 42.225)	38.8 (35.3, 43.1)	0.987
Creatinine (mg/dL)	0.9 (0.7, 1)	0.9 (0.725, 1.2)	1 (0.8, 1.2)	0.9 (0.7, 1.1)	0.082
Bun (mg/dL)	16 (11, 20)	16 (12, 20)	17 (13, 23)	19 (13, 26)	0.060
Chloride (mmol/L)	104 (101, 106)	104 (101, 106)	102 (100, 105)	104 (101, 107)	0.058
Glucose (mg/dL)	123 (99, 150)	125 (107.25, 156.5)	133 (116, 158)	130 (109, 164)	0.072
Sodium (mmol/L)	139 (137, 142)	140 (138, 142)	139 (137, 141)	139 (138, 142)	0.561
Potassium (mmol/L)	3.9 (3.6, 4.1)	3.9 (3.6, 4.2)	3.9 (3.6, 4.2)	4 (3.6, 4.3)	0.786
Anion gap (mmol/L)	14 (12, 16)	15 (13, 16)	16 (14, 18)	16 (13, 18)	**0.003**
Bicarbonate (mmol/L)	23 (22, 26)	24.5 (23, 26)	24 (22, 25.75)	23 (20, 25)	**0.001**
Calcium (mg/dL)	9 (8.6, 9.3)	8.9 (8.6, 9.3)	8.8 (8.5, 9.075)	8.7 (8.3, 9)	**0.001**
Albumin (g/dL)	4 (3.6, 4.2)	3.9 (3.6, 4.2)	3.8 (3.4, 4.175)	3.7 (3.2, 4.1)	**0.019**
*Events*					
28-day mortality	11 (2.7%)	11 (2.7%)	24 (5.9%)	33 (8.1%)	**<0.001**

LDH (U/L): Q1(<194.7) Q2(194.7∼233.0) Q3(233.0∼287.3) Q4(>287.25). LDH: Lactate dehydrogenase; APS III: Acute physiology score III; SAPS II: Simplified acute physiological score II; GCS: Glasgow coma scale; WBC: White blood cell; RBC: Red blood cell; MCH: Mean corpuscular hemoglobin; MCHC: Mean corpuscular hemoglobin concentration; MCV: Mean corpuscular volume; RDW: Red blood cell distribution width; Bun: Blood urea nitrogen.

**Table 2:** Demographic and clinical characteristics of patients with non-traumatic ICH

**Variables**	**28-day mortality,** ***n* ═ 79**	**28-day survival,** ***n* ═ 327**	**t/Z /χ^2^** **value**	***P* value**
Age (years)	71 (62, 83 )	68 (55, 78)	−2.744	0.006
Female, *n* (%)	32 (7.9%)	132 (32.5%)	0.000	0.981
Race, *n* (%)			9.706	0.021
White	37 (9.1%)	194 (47.8%)		
Yellow	0 (0%)	8 (2%)		
Black	7 (1.7%)	35 (8.6%)		
Other	35 (8.6%)	90 (22.2%)		
*Severity score*				
Charlson index	7 (5,8)	6 (4,8)	−2.817	0.005
SAPS II	39 (35,48)	30 (23,37)	−7.189	<0.001
APS III	55 (42,80)	40 (29,52)	−5.990	<0.001
GCS	15 (14,15)	15 (14,15)	−0.519	0.604
*Commorbidities, n (%)*				
Myocardia infarct	9 (2.2%)	16 (3.9%)	3.594	0.058
Congestive heart failure	12 (3%)	37 (9.1%)	0.900	0.342
Chronic pulmonary disease	13 (3.2%)	38 (9.4%)	1.354	0.244
Mild liver disease	9 (2.2%)	17 (4.2%)	4.072	0.043
Diabetes	7 (1.7%)	16 (3.9%)	1.206	0.272
*Laboratory tests*				
RBC (10^12^/L)	4.099 ± 0.712	4.377 ± 0.709	3.144	0.002
WBC (10^9^/L)	11.0 (8.1, 13.8)	9.6 (7.5, 12.3)	−2.139	0.032
Platelet (K/µL)	194 (133, 261)	214 (173, 270)	−1.874	0.061
MCH (pg)	30.7 (29.2, 32.2)	30.2 (28.6, 31.8)	−1.516	0.130
MCHC (g/L)	33.081 ± 1.613	33.410 ± 1.498	1.725	0.085
MCV (fL)	92 (88, 98)	90 (86, 94)	−2.558	0.011
RDW (%)	13.9 (13.3, 15.2)	13.5 (13.1, 14.3)	−2.369	0.018
Hemoglobin (g/dL)	12.5 (11.2, 13.6 )	13.2 (11.9, 14.5 )	−2.779	0.005
Hematocrit (%)	37.377 ± 5.6852	39.184 ± 5.453	2.621	0.009
Creatinine (mg/dL)	1.0 (0.8, 1.4)	0.9 (0.7, 1.1)	−1.800	0.072
Bun (mg/dL)	18.0 (13.0, 24.0)	16.0 (12.0, 22.0)	−2.292	0.022
Chloride (mmol/L)	103.0 (99.0,107.0)	103.0 (101.0,106.0)	−0.435	0.663
Glucose (mg/dL)	142.0 (113.0, 167.0)	126.0 (106.0, 153.0)	−1.906	0.057
Sodium (mmol/L)	139.0 (138.0, 142.0)	139.0 (137.0, 142.0)	−0.765	0.444
Potassium (mmol/L)	3.9 (3.5, 4.2)	4.0 (3.7, 4.0)	−2.582	0.010
Anion gap (mmol/L)	16.0 (13.0,18.0)	15.0 (13.0, 17.0)	−1.839	0.066
Bicarbonate (mmol/L)	23.0 (21.0, 25.0)	24.0 (22.0, 26.0)	−1.764	0.078
Calcium (mg/dL)	8.7 (8.3, 9.0)	8.9 (8.5,9.2)	−2.987	0.003
Albumin (g/dL)	3.6 (3.2,4.0)	3.9 (3.5,4.2)	−3.718	<0.001
LDH (U/L)	280 (230,346)	227 (185,279)	−4.915	<0.001

APS III: Acute physiology score III; SAPS II: Simplified acute physiological score II; GCS: Glasgow coma scale; WBC: White blood cell; RBC: Red blood cell; MCH: Mean corpuscular hemoglobin; MCHC: Mean corpuscular hemoglobin concentration; MCV: Mean corpuscular volume; RDW: Red blood cell distribution width; LDH: Lactate dehydrogenase; ICH: Intracerebral hemorrhage; Bun: Blood urea nitrogen.

### K-M curves

**Figure 2.**
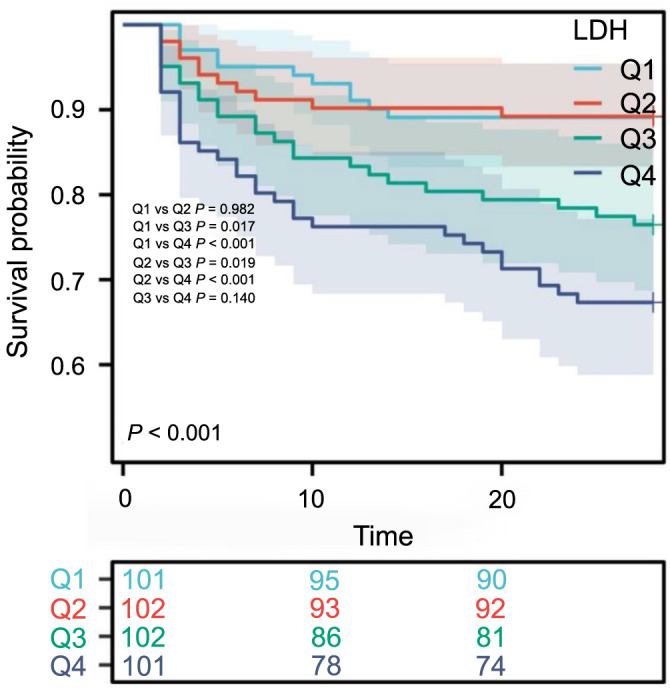
**Cumulative incidence and Kaplan–Meier survival analysis curves for all-cause mortality.** LDH (U/L): Q1 (<194.7) Q2 (194.7∼233.0) Q3 (233.0∼287.3) Q4 (>287.25). X-axis: Survival time (days). Y-axis: Survival probability. LDH: Lactate dehydrogenase.

K-M survival curves illustrated the 28-day all-cause mortality rates among groups categorized by LDH quartiles. The ICU survival rate of patients in the Q4 group (LDH > 287.25) was lower compared to those in the Q1 group (LDH < 194.7) and the Q2 group (194.7 < LDH < 233.0), who had lower LDH levels (*P* < 0.001). However, there was no significant difference compared to Q3 (*P* ═ 0.140) (Figure 2).

### Multivariate Cox regression analysis

The relationship between LDH and 28-day all-cause mortality was examined using a Cox proportional hazards regression model, as shown in Table 3. Patients in the highest LDH quartile were significantly associated with an increased risk of death across three Cox proportional hazards models: the unadjusted model [HR, 3.401 (95% CI 1.719–6.731), *P* < 0.001], the partly adjusted model [HR, 2.422 (95% CI 1.211–4.846), *P* ═ 0.012], and the fully adjusted model [HR, 3.054 (95% CI 1.522–6.126), *P* ═ 0.002], compared to subjects in the lowest quartile. This association demonstrated a trend of increasing risk with higher LDH levels (*P* for trend < 0.01). The results of the Cox proportional hazards regression analysis were consistent with those obtained from the K-M curve analysis.

**Table 3:** Cox proportional hazard models for 28-day all-cause mortality

**Groups**	**Model 1**		**Model 2**		**Model 3**	
	**HR (95% CI)**	***P* value**	**HR (95% CI)**	***P* value**	**HR (95% CI)**	***P* value**
*28-day mortality*						
Q1	Ref		Ref		Ref	
Q2	1.004 (0.432–2.316)	0.992	0.855 (0.369–1.982)	0.715	1.039 (0.449–2.4)	0.930
Q3	2.306 (1.130–4.708)	0.022	1.75 (0.845–3.626)	0.132	2.006 (0.976–4.124)	0.058
Q4	3.401 (1.719–6.731)	<0.001	2.422 (1.211–4.846)	0.012	3.054 (1.522–6.126)	0.002
*P* for trend		<0.001		<0.001		<0.001

Model 1: Unadjusted. Model 2: Adjusted for mild liver disease; Charlson comorbidity index; Saps ii; Aps iii. Model 3: Adjusted for age; Rbc; Hemoglobin; Wbc; Hematocrit; Mcv; Rdw; Creatinine; Bun; Glucose; Potassium; Aniongap; Bicarbonate; Calcium; Albumin; Charlson comorbidity index; Sapsii; Aps iii. HR: Hazard ratio; CI: Confidence interval; Ref: Reference.

### Nonlinear analysis

**Figure 3.**
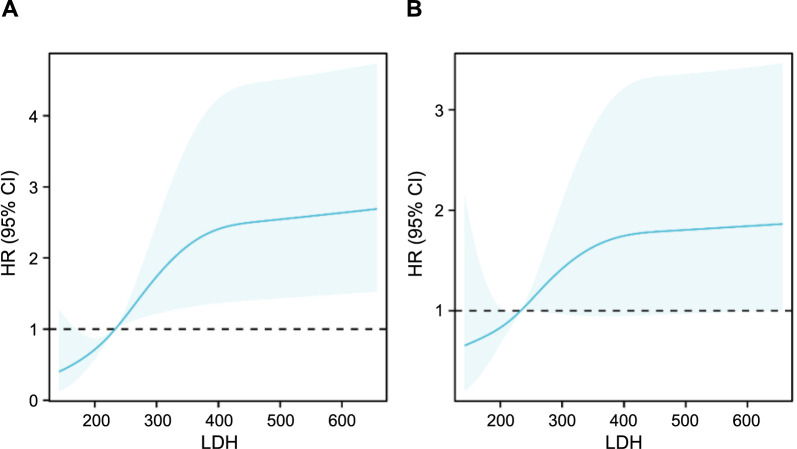
**Restricted cubic spline curve for the LDH hazard ratio. Heavy central lines represent the estimated adjusted hazard ratios, with shaded ribbons denoting 95% confidence intervals.** (A) Unadjusted. (B) Adjusted for age; Rbc; Hemoglobin; Wbc; Hematocrit; Mcv; Rdw; Creatinine; Bun; Glucose; Potassium; Aniongap; Bicarbonate; Calcium; Albumin; Charlson comorbidity index; Sapsii; Aps iii. LDH: Lactate dehydrogenase; HR: Hazard ratio; CI: Confidence interval.

RCS models demonstrated an L-shaped relationship between LDH levels and the 28-day all-cause mortality rate, indicating a nonlinear association (Figure 3).

### Subgroup analysis

**Figure 4.**
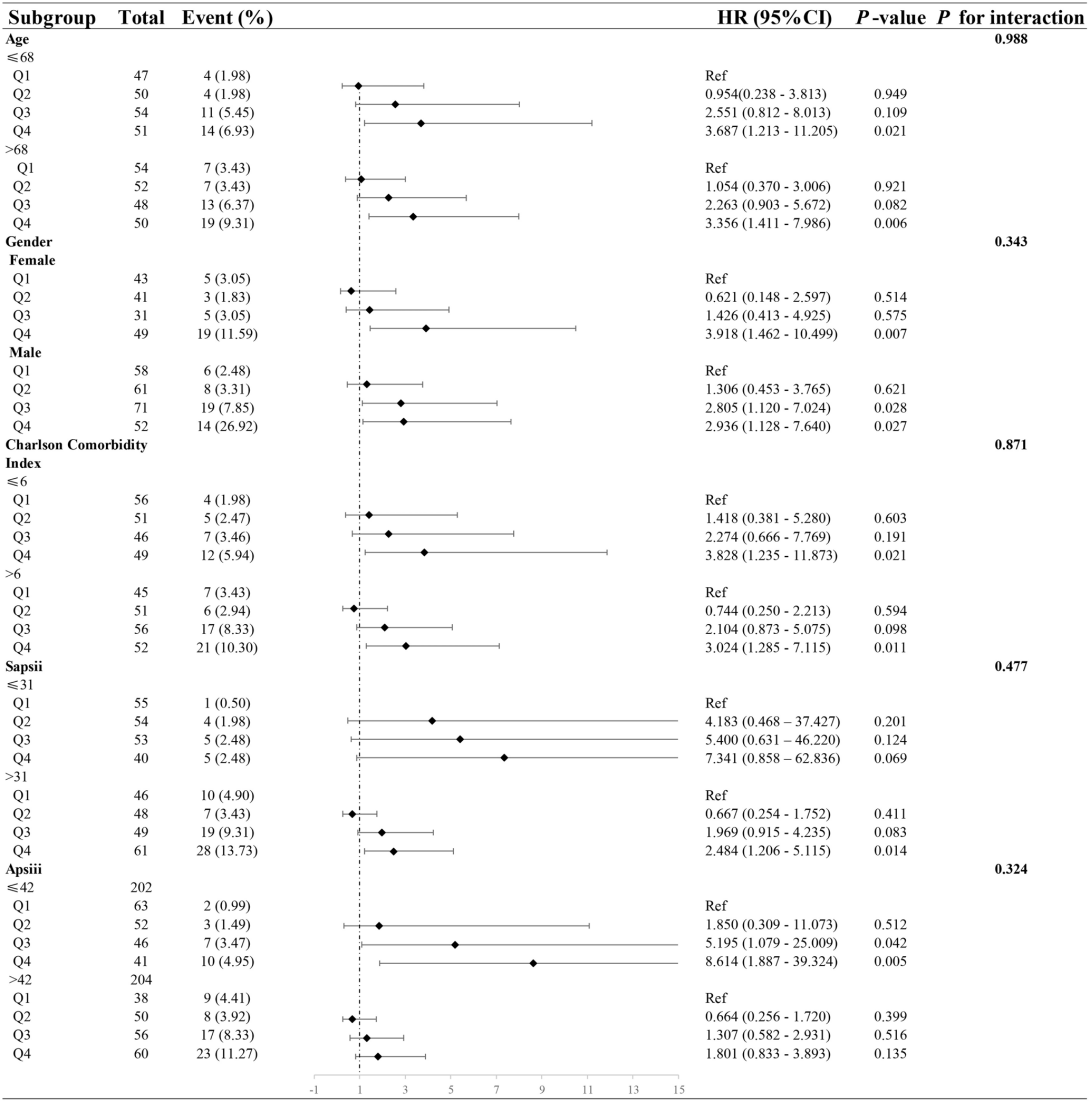
**Subgroup analysis for the correlation between LDH and 28-day all-cause mortality.** LDH: Lactate dehydrogenase; HR: Hazard ratio; CI: Confidence interval; Ref: Reference.

Given the potential influence of various patient subgroups on outcomes, we investigated the impact of LDH levels within stratified subgroups. Subgroup analyses were conducted based on age, gender, Charlson Comorbidity Index, SAPS II, and APS III scores (Figure 4). The results demonstrated that the association between LDH levels and 28-day all-cause mortality was consistent across various age and gender subgroups. In the age subgroup, patients under 65 years [HR, 3.7; 95% CI, 1.213–11.205; *P* ═ 0.021] and over 65 years [HR, 3.356; 95% CI, 1.411–7.986; *P* ═ 0.006] both showed significant associations. Similarly, for gender subgroups, significant associations were observed in males [HR, 2.936; 95% CI, 1.213–11.205; *P* ═ 0.027] and females [HR, 3.918; 95% CI, 1.462–10.499; *P* ═ 0.007]. For the Charlson Comorbidity Index subgroup, patients with scores ≤6 [HR, 3.828; 95% CI, 1.235–11.873; *P* ═ 0.021] and scores >6 [HR, 3.024; 95% CI, 1.285–7.15; *P* ═ 0.011] also showed significant correlations with LDH levels. No significant differences were detected in analyses by age, gender, Charlson Comorbidity Index, SAPS II, and APS III scores, suggesting that the subgroup analysis was robust and minimally influenced by confounding factors (all *P* for interaction > 0.05).

## Discussion

To the best of our knowledge, no published studies have compared the mortality predictive value of LDH in non-traumatic ICH. The main finding of our study is that elevated LDH levels in hospitalized patients with non-traumatic ICH are associated with an increased 28-day mortality rate. This association remained robust even after adjusting for various clinical and laboratory variables. Our results extend the application of LDH to cerebrovascular disease, highlighting its potential value as a decision-making tool for clinicians managing patients with non-traumatic ICH.

ICH is a relatively common stroke syndrome, accounting for approximately 15% of all strokes and 50% of stroke-related mortality, which equates to approximately 2.8 million deaths worldwide each year [7] ICH disrupts brain fluid homeostasis. In addition to the initial hematoma mass, ICH leads to blood–brain barrier disruption and parenchymal cell swelling, resulting in brain edema and intracranial hypertension, which affect patient prognosis [8]. Therapeutic interventions for acute ICH have focused on arresting hemorrhage expansion, reducing clot volume in both intraventricular and parenchymal hematomas, and targeting perihematomal edema and inflammation [9]. Despite significant progress in acute management, the optimal surgical approach remains to be determined [10]. Multiple indicators currently predict the severity of ICH. The entry of peripheral WBC into the central nervous system may indicate a more severe inflammatory response, while elevated blood glucose levels may exacerbate brain edema through oxidative stress, leading to more severe neurological deficits [11]. However, relevant predictive factors for survival rates are not well established. Given the high incidence and mortality of this disease, predicting survival through objective laboratory indicators after onset could facilitate early intervention and significantly benefit clinical treatment.

LDH is an enzyme widely distributed across various cells and tissues, including muscle, liver, and brain. It is released into the peripheral blood following cellular damage and is commonly used as a diagnostic biomarker for diseases and tissue injury [2]. It is also recognized as a central metabolite in brain physiology [12]. There is a significant correlation between serum LDH and lactate levels, both of which reflect the extent of tissue damage [13, 14]. LDH is involved in the lactate-pyruvate metabolism cycle, influencing the production of reactive oxygen species (ROS). Increased ROS can lead to oxidative stress, contributing to cell death and worsening neurological deficits post-ICH [15]. LDH not only correlates with clinical prognostic indicators but also guides subsequent therapeutic interventions [16]. Recent studies suggest that inhibiting LDH may have neuroprotective effects, potentially reducing the extent of brain injury post-ICH. Targeting LDH activity could be a novel strategy for managing secondary brain injury following hemorrhagic stroke [17].

ICH can induce a cascade of secondary injury mechanisms, including excitotoxicity, oxidative stress, and inflammation. LDH may serve as a biomarker for these processes, indicating the severity of brain injury and the metabolic state of the affected tissue [18]. Multiple studies have demonstrated that elevated levels of LDH are associated with the severity of various diseases and an increased risk of mortality. These include sepsis, postoperative pneumonia in patients with aneurysmal subarachnoid hemorrhage, and acute kidney injury, among others [19, 20]. Chu et al. [2] demonstrated that LDH is a reliable predictor of early hematoma expansion and poor outcomes in patients with ICH. Collectively, these studies suggest the potential significance of the association between LDH levels and clinical outcomes in critically ill patients. Zhang et al. [21] demonstrated that LDH levels are associated with increased 30- and 90-day mortality, as well as in-hospital mortality, in patients with ARDS. Yan et al. [22] demonstrated that high LDH levels are associated with an increased risk of stroke-associated pneumonia (SAP) in patients with acute ischemic stroke (AIS). Recent studies have found that LDH silencing significantly reduces angiogenesis, suggesting that LDH plays a crucial role in maintaining vascular homeostasis [23]. Koguchi-Yoshioka et al. [24] demonstrated that psoriatic patients with high serum LDH levels may benefit from apremilast.

Our results suggest an association between elevated LDH levels and the severity and outcomes of ICH. In patients with ICH, secondary neuroinflammatory reactions have been observed, and neuroinflammation can interact in a feedback loop, leading to both positive and negative effects and resulting in varying degrees of systemic symptoms. Therefore, LDH levels, which are influenced by neuroinflammation, may predict the severity of ICH and, consequently, mortality rates. However, the precise biological mechanisms underlying the relationship between LDH and non-traumatic ICH remain unclear.

Our study has several limitations. First, as a retrospective analysis, it is subject to confounding bias despite controlling for various factors and performing subgroup analyses. Second, we measured blood samples only upon admission and did not account for fluctuations in LDH levels during hospitalization. Third, as the study relies on a database, there may be issues with data quality, such as incompleteness or inaccuracies. Further research is needed to elucidate the key mechanisms by which LDH affects patients with non-traumatic ICH.

## Conclusion

Our retrospective analysis indicates that elevated LDH levels are associated with a progressive increase in the 28-day all-cause mortality rate among patients with non-traumatic ICH. These findings may provide healthcare professionals with a valuable tool for early intervention and clinical planning to improve patient outcomes. Further validation of LDH as a readily available and objective biomarker is required through large-scale, multicenter prospective studies.

## Data Availability

In this study, data from public databases were analyzed. These data can be found here: MIMIC-IV: https://www.physionet.org/content/mimiciv/2.2/.
